# Targeted DNA oxidation and trajectory of radical DNA using DFT based QM/MM dynamics

**DOI:** 10.1093/nar/gkz089

**Published:** 2019-02-18

**Authors:** Pradip K Biswas, Sandipan Chakraborty

**Affiliations:** Laboratory of Computational Biophysics and Bioengineering, Department of Physics, Tougaloo College, Tougaloo, MS 39174, USA

## Abstract

Molecular insight into electronic rearrangements and structural trajectories arising from oxidative damages to DNA backbone is of crucial importance in understanding the effect of ionizing radiation, developing DNA biosensors and designing effective DNA cleaving molecules. Employing a Density Functional Theory based multi-scale Quantum-Mechanical-Molecular-Mechanical (QM/MM) simulation and a suitable partitioning of the Hamiltonian on solvated nucleotide, and single-, and double-stranded DNA, we mimic hydrogen transfer reactions from the backbone by OH radicals and report structural trajectories arising from on-the-fly electronic charge- and spin-density redistribution in these three different structural topologies of DNA. Trajectories reveal that H4′ abstraction can disrupt the deoxyribose moiety through the formation of C4′=O4′ ketone and a π-bond with base at C1′-N9 in a nucleotide versus only partial ketone formation in single- and double-stranded DNA, where the orientation of the base is topologically restrained. However, H5′ abstraction can lead DNA cleavage at 5′ end through the formation of C5′=O5′ ketone and breakage of P-O5′ bond. Results demonstrate that structural damages from oxidative reactions are restrained by base stacking and base-pair hydrogen bonding. The methodology can be suitably used to study targeted DNA and RNA damages from radicals and radiomimetic drugs to design DNA cleaving molecules for chemotherapy.

## INTRODUCTION

Free radicals of cellular or external origin (e.g. radiation induced) and radiomimetic drugs can cause oxidative damage to cellular DNA and RNA (1–3). The oxidation of ribose or deoxyribose is believed to lead to the dissociation of sugar-phosphate bonds and eventual scission of DNA strands (4–6). These DNA lesions can be self-repaired or otherwise lead to mutagenesis, carcinogenesis, aging, inherited disease and cell death (1,7–9). To understand oxidative damage to DNA and strand scission (1,7–13) or to develop synthetic molecules for DNA cleavage (3,14,15) or DNA biosensors, it is essential to identify the short-lived intermediaries considering the structural constraints present in a single- or double-stranded DNA in physiologically relevant conditions. This presents an enormous challenge for computational chemists and also it is difficult to identify the short-lived and chemically similar intermediaries in experimental set-up. Experimental techniques, like GC-MS (gas chromatography-mass spectrometry), gel electrophoresis, transient photocurrent method and the combination of autoradiography with high-resolution polyacrylamide gel electrophoresis (PAGE), provide crucial insights into the molecular mechanism of nucleic acid damage (16–21). However, as unraveling the chemical pathways of a specific DNA damage from an oxidative interaction of DNA in solution still remained elusive to track, model DNA nucleotide compounds have been used to glean into the reaction trajectory (10–12). Here, we describe a Density Functional Theory (DFT) based multi-scale Quantum-Mechanical-Molecular-Mechanical (QM/MM) simulation technique to elucidate the structural trajectory of oxidative DNA damage pathways.

In order to understand the structural landscape of the oxidative reaction pathways, it is essential to corroborate the experimentally known final states of an oxidative reaction with predicted intermediate steps. This can suitably be done with an appropriate molecular dynamics simulation on a realistic DNA system (with water and in physiological temperature and pressure with appropriate ionic strength). However, owing to the large size of a realistic DNA system, it is impractical to employ an *ab initio* quantum theory to the whole system that is required to understand the resulting electronic rearrangement during and after a hydrogen abstraction. *Ab initio* theoretical studies, thus, mostly focus on evaluating energetics of various perceived intermediate states and infer about the possible reaction pathways (22–26). Using Monte Carlo simulations, Spotheim-Maurizot *et al.* found that for hydrogen abstraction from double-stranded DNA the most probable sites are the H4′ and H5′ positions (27). Recently, reactive molecular dynamics using ReaxFF potential has been used to explore the effect of oxidative stress on DNA caused by OH radicals. However, electronic rearrangements during the process are completely ignored (28). The combined QM/MM simulation protocol (29–36), however, offers a suitable tool to address the electronic rearrangement of the chemically active site of a large system and address the formation and breaking of chemical bonds while taking into account the effect of the surrounding medium. This has been successfully employed in studying hydrogen transfer from guanine (37) and daidzein (38).

The QM/MM method uses partitioning of the Hamiltonian of a large supramolecular system into two interacting subsystems with a small and tractable chemically active part treated in QM, and the rest of the system treated in MM. The effect of MM on QM is taken as an electrostatic perturbation of the QM Hamiltonian, and the effect of QM on MM as electrostatic forces on MM atoms. The QM/MM method has been successfully employed previously in various systems including photoinduced damage of DNA and RNA nucleobases in gas and bulk phases (35), mimic the effect of methylation and hydroxylation on a guanine base of a dodecameric B-DNA in solution (39) and in estimating the effect of solvent on the hydrogen abstraction from a guanine base in a nucleotide (37,40) and also to explore OH radical scavenging mechanism of an antioxidant, daidzein (38). Here, we report the structural trajectories of oxidative DNA damage by OH-radical induced hydrogen abstraction using QM/MM dynamics. Though there are numerous studies aimed at drawing conclusions about strand break using model nucleotide compounds, there is no study that systematically investigate any specific role of the attached base, base stacking and base-pair hydrogen bonding in an oxidative damage to DNA backbone. Here, in order to specifically understand these roles as arising from the structural topology, we employ three different levels of a DNA system—a complete nucleotide, a single-stranded and a double-stranded DNA all solvated in water. Our results reveal that the structural landscape of oxidative DNA damage due to backbone hydrogen abstraction is significantly restrained by base stacking in a single-stranded DNA and by base stacking and base-pair hydrogen bonding in a double-stranded DNA. Present study suitably demonstrates the applicability of the QM/MM technique to study radical-induced DNA and RNA damage reaction pathways at molecular level.

## MATERIALS AND METHODS

### System preparation and equilibration

We use a 12 base-paired (24 bases altogether) DNA crystal structure (PDB ID: 424D) to construct the simulating systems. A single nucleotide, single-stranded DNA and double-stranded DNA systems with OH radicals and in SPC explicit water were then prepared. For all the three cases, we choose the guanosine at the seventh position (near the center) to target. We choose guanine because it has the largest base-pair stacking energy and the most stable one compared to the adenine, thymine and cytosine, thus providing the maximum restraints to structural damage. Here we initially targeted the H4′ hydrogen since it is the most accessible site by water. We then selected a water molecule nearest to the H4′ hydrogen and converted it into an OH radical by deleting a hydrogen atom. At this point, to obtain a preliminary system suitable to study the effect of hydrogen abstraction, we manually docked the OH radical near our target site using VMD (41) such that the distance between H4′ and radical oxygen should be ∼2 Å. Similarly, we also prepared a double-stranded DNA with docked OH radical targeting H5′ hydrogen to study the structural dynamics of hydrogen atom transfer reaction from H5′ site. All the systems were solvated using SPC explicit water model (42). The box size was such that the minimal distance between DNA and box edge is 10 Å. Appropriate numbers of NA^+^ ions were added to make the system charge neutral. All the equilibrium simulations were performed using OPLS force field (43,44) using GROMACS packages (45–47). We then provided a short energy minimization of the system by restraining the positions of the DNA and OH to allow the water around DNA and OH to settle down. We then equilibrate the whole system at 300 K by performing a position-restrained dynamics as implemented in GROMACS where the DNA and the OH are position-restrained. From the position-restrained dynamics trajectory, we select a frame where the OH radical is in close proximity to the hydrogen abstraction site and also was in a suitable orientation as hydrogen abstraction does depend on the H′-•OH angle as well. This is also to mention here that, as noted by Chalmet and Ruiz-López (48), the Lennard Jones parameters assigned to the hydrogen atoms of the DNA backbone in OPLS-AA are found to generate strong repulsion. Thus, while preparing the systems during equilibration with Molecular Dynamics, the OH radical diffuses away. To address this problem, we kept the Lennard Jones interactions between the hydrogen of the backbone and OH to zero as has also been done by Chalmet and Ruiz-López (48). To check whether this adjustment to the hydrogen atom LJ parameters can affect the DNA structural stability, we performed MD simulations on solvated DNA with and without the changes in the LJ parameter and found that this change has no impact on DNA structural stability. In addition, during QM/MM dynamics, the default parameters of OPLS force field were used as this adjustment was not necessary for the QM Hamiltonian since both the OH and the DNA active site are treated in QM that completely disregards their assigned classical parameters.

### QM/MM dynamics

For the combined QM/MM simulation, we use the GROMACS-CPMD QM/MM code (33) that couples the molecular mechanical code GROMACS (45–47) and the density functional theory based quantum mechanical code CPMD (http://www.cpmd.org/). To describe the QM subsystem, we use the norm conserving ultrasoft Vanderbilt pseudo-potentials with an energy cut of 25 Rydberg (Ry) (49), a local spin density approximation (LSDA), and Becke (50) exchange and Lee-Yang-Parr (BLYP) gradient-corrected functional (51). For the wave function minimization in CPMD, we use the Poisson solver of Martyna and Tuckerman (52). For the classical subsystem, we use the explicit SPC water model for solvent together with the OPLS force field for the nucleic acids as imported and developed from AMBER (53) by the RNP group (http://rnp-group.genebee.msu.su/3d/ff.htm). In GROMACS-CPMD QM/MM, the MM atom partial charges are given a Slater type expansion to make their interaction with the QM subsystem as realistic as possible. In GROMACS-CPMD QM/MM, GROMACS controls the molecular dynamics simulation and CPMD provides the optimized wave function for the QM subsystem appropriately perturbed by the MM subsystem. For our QM/MM, we have performed a constant pressure and temperature (NPT) MD simulation with a reference temperature of 300 K. For the QM subsystem, cubic cells of size of (i) (21.17 Å × 18.52 Å × 15.88 Å), (ii) (18.52 Å × 23.81 Å × 23.81 Å) and (iii) (18.52 Å × 23.81 Å × 23.81 Å) were used for the single nucleotide, single strand and double strand, respectively. In essence, while considering a QM box size, we have kept a minimum margin of 5 Å in all six sides of the QM system. As GROMACS-CPMD molecular dynamics (33) is controlled by GROMACS (45) and CPMD (54) provides electronic density minimization, our QM subsystem essentially follows a Born-Oppenheimer dynamics of GROMACS. This frees us from using ultra-low time step required for Car-Parrinelo Molecular Dynamics (54) that explicitly includes the electronic degrees of freedom into the Lagrangian. However, the QM time-step is still crucial for a hybrid QM/MM simulation as the QM subsystem will not have bond-constraints and could lead to ultra large kinetic energies of the hydrogen atoms in the QM subsystem. This restricts our maximum time step to 1.0 fs as recommended in GROMACS without any bond constraints. To test the suitability of the time steps, we ran the double-stranded system at four time steps: 0.5, 0.75, 1.0 and 1.5 fs and found 1.0 fs provides the optimum step suitable for our case.

## RESULTS

**Figure 1.**
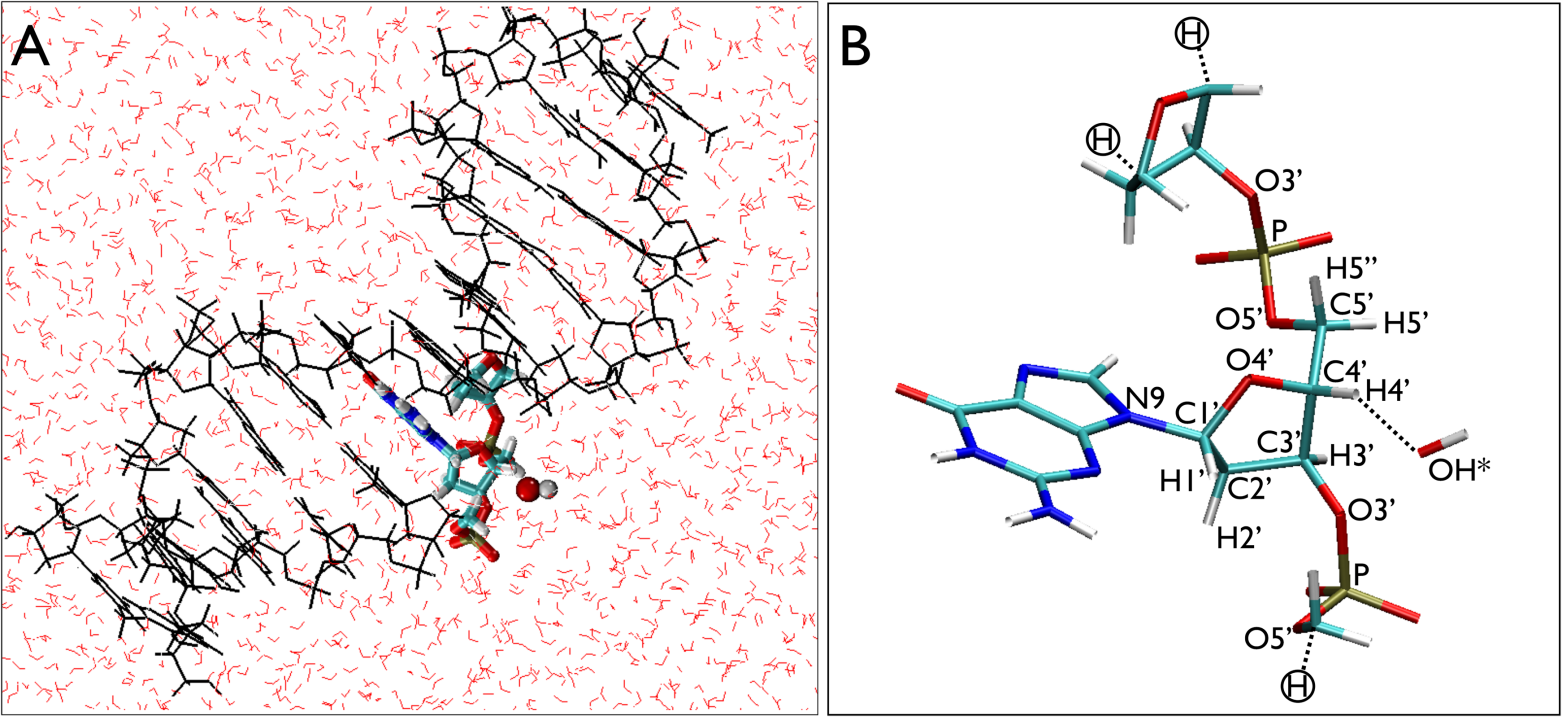
(**A**) A 12-bp double-stranded DNA (PDB-ID: 424D.pdb) and an OH radical in solution. An overview of the complete system with QM/MM partitions. The highlighted portion has been treated in QM and the rest of the system is treated in MM. (**B**) The atomic nomenclature used for the QM subsystem is shown together with the linker hydrogen (circled) used to saturate the truncated bonds.

Our primary objective was to architect an effective theoretical model to study the molecular mechanism of DNA damage originated from hydrogen abstractions by radicals. For this, we employed a hybrid QM/MM simulation protocol on three different systems: (i) a single nucleotide, (ii) a single-stranded DNA and (iii) a double-stranded DNA; all solvated in water and an OH radical targeted to the active site. We choose the OH radical as this is a known byproduct of secondary effect of ionizing radiation in water that can potentially damage the DNA. Also, we mainly target the abstraction of the H4′ hydrogen of the sugar moiety of DNA backbone as it is one of the most exposed sites in water (exhibited in Figure 1A). Nevertheless, the study can be extended to target any other site of a DNA or of a different system together with any other radical or oxidant. To exemplify, we also present a study of H5′ hydrogen abstraction from a double-stranded DNA solvated in water. A schematic representation of the starting configuration of a double-stranded DNA with a nearby OH radical is shown in Figure 1A and the positional nomenclature used in this study has been summarized in Figure 1B.

### Optimization of a QM subsystem

**Figure 2.**
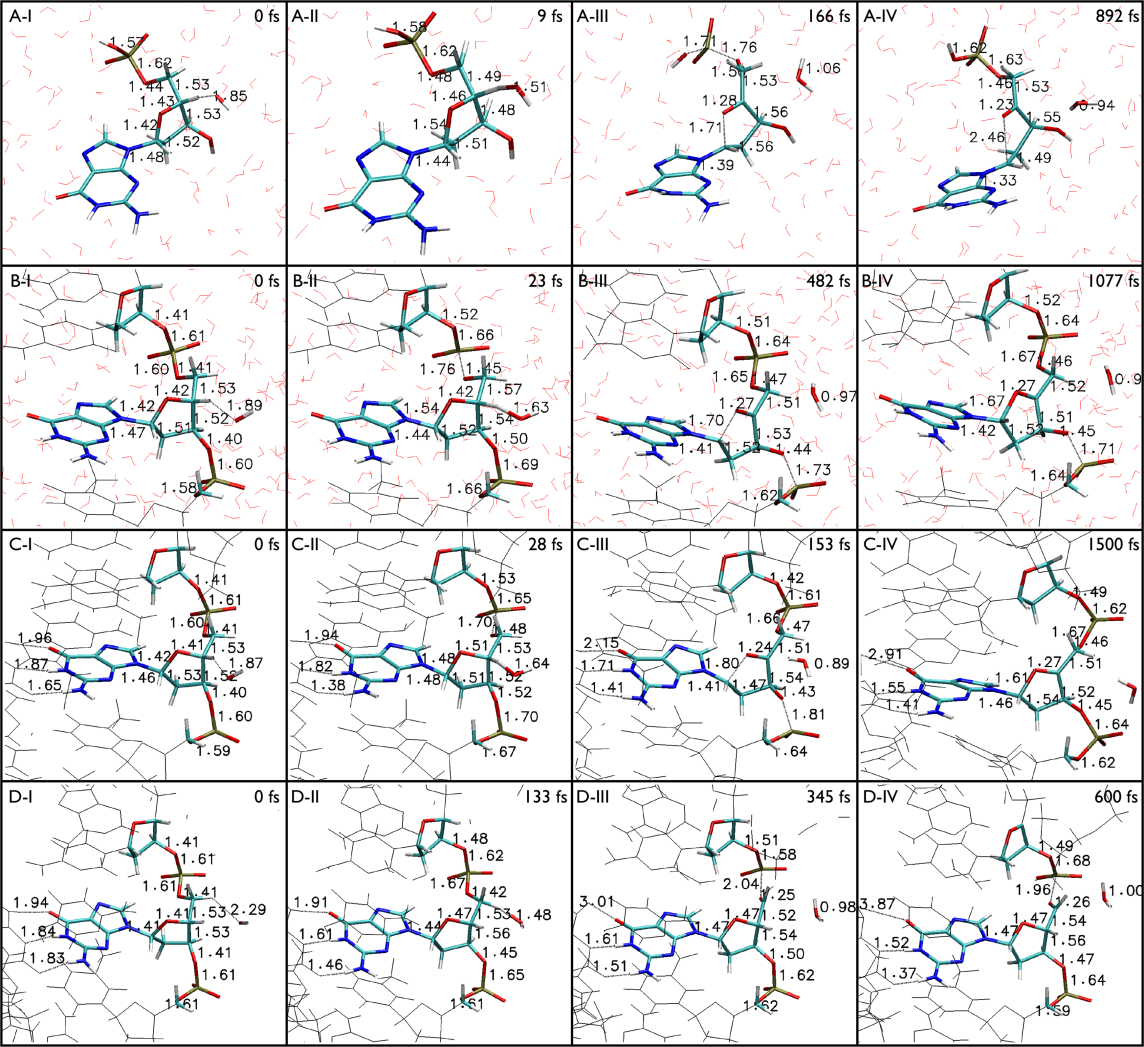
Selected frames from QM/MM dynamics of oxidative DNA damage by OH radical. (**A**) H4′ abstraction from Deoxyguanosine Monophosphate, (**B**) H4′ abstraction from single-stranded DNA, (**C**) H4′ abstraction from double-stranded DNA and (D) H5′ abstraction from double-stranded DNA.

Before discussing our results, we focus on a suitable size of a QM subsystem that is crucial for a QM/MM partition of the active site from the whole system. During our trial runs with the simple DNA nucleotide system, we found that it is crucial to have a minimum size of the QM subsystem that is large enough to be able to fully absorb the electronic charge density redistribution arising out of a chemical reaction (here hydrogen abstraction) but small enough to reduce the computational cost. For the nucleotide, when we took the phosphate, the deoxyribose, and the OH in the QM subsystem leaving the attached base and the solvent water to be treated by MM, we found that the H4′ abstraction does not have the expected effect of the formation of a ketone at C4′-O4′ as C1' is now restricted to have any electronic rearrangement with the base since N9 is partitioned into the classical MM system. However, as soon as we included the guanine into the QM subsystem, we found an increased electron sharing between C1′ and N9 resulting in a stronger C1′-N9 bond and a reduced electron density across the C1′-O4′ bond suggesting electronic rearrangement between the deoxyribose and the base. Similarly, we have also found that for studying H4′ hydrogen abstraction from single- or double-stranded DNA, we need the corresponding DNA nucleotide, the 3′-end deoxyribose and the 5′ end phosphate together with the attached C5′ carbon and H5′ hydrogen from the adjacent residues (as shown in Figures 1B, 2B, and 2C).

**Figure 3.**
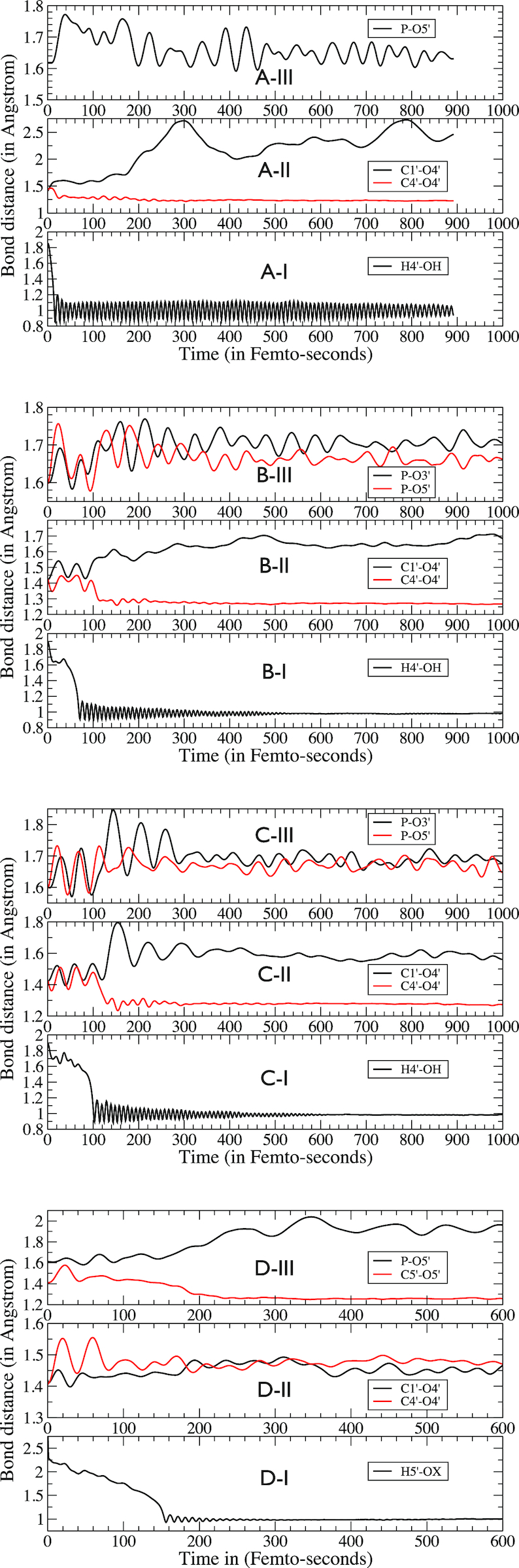
QM/MM dynamics of selected bond lengths for H4′ abstraction from (**A**) dGMP (Deoxyguanosine Monophosphate), (**B**) single-stranded DNA, (**C**) double-stranded DNA and (**D**) H5′ abstraction from double stranded DNA.

In Figure 2, we provide selected frames from the QM/MM simulation dynamics that exhibit H4′ hydrogen abstraction by OH radical from (i) end-capped nucleotide (2A), (ii) single-stranded DNA (2B), (iii) double-stranded DNA (2C) and (iv) H5′ hydrogen abstraction by OH radical from double-stranded DNA (2D) and subsequent DNA structural changes. Here, we have monitored about 12 bonds (Figure 3), the bonds that are of key interest are: C4′-O4′, C1′-O4′, C1′-N9, P-O3′ and P-O5′. Among these, the first three are particularly related to the damage of the deoxyribose ring and the last two are related to the cleavage of the DNA.

### H4′ abstraction from DNA nucleotide

**Figure 4.**
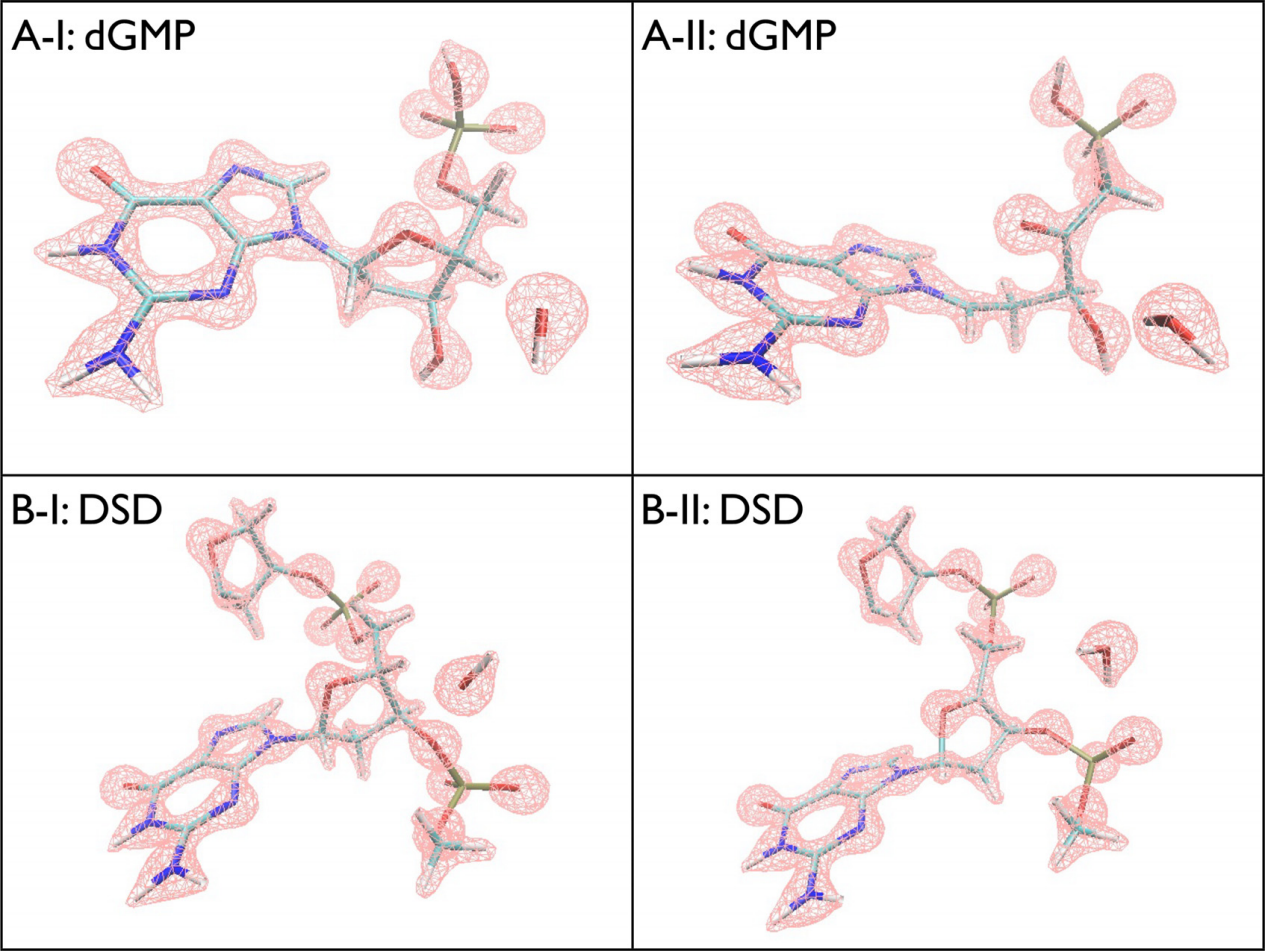
Electron density profile from H4′ hydrogen abstraction dynamics of (**A**) dGMP (Deoxyguanosine Monophosphate) and (**B**) DSD (Double Stranded DNA).

In Figure 2A, we display selected frames from the trajectory of H4′ hydrogen abstraction from C4′ and subsequent structural changes in the radical nucleotide. As C4′ loses its chemical bonding with H4′, it tries to form an additional bonding with O4′ for its unpaired electron. Figure 2A-I–IV and Figure 3A-I–III show some intermediate frames of how C4′ forms an additional bond with O4′ leading to the formation of a ketone (C4′ = O4′) and the disruption of the C1′-O4′ bond. The C4′-O4′ bond distance reduces from 1.42 to 1.23 Å, while the C1′-O4′ bond distance increases rapidly just after the hydrogen abstraction is complete from 1.42 to 2.46 Å, a completely broken bond. However this couldn’t happen without C1′ loosing bond with O4′ electron by sharing additional electronic charge densities from its neighbor; in the present case, this additional electron sharing is coming from the nitrogen (N9) of the guanine base. We observed the C1′-N9 bond distance decreases from 1.48 to 1.33 Å. Figure 3A provides an insight to some crucial bond distances over time during this reaction. As in Figure 3A-I, the H4′-OH distance decreases rapidly ∼10 fs indicating the completion of H4′ hydrogen capture by radical OH. Figure 3A-II exhibits the behavior of the C4′-O4′ and C1′-O4′ bond distances. The C4′-O4′ bond starts decreasing while the C1′-O4′ bond distance starts increasing immediately after the dissociation of H4′ leading to the processes of a ketone formation (C4′ = O4′) and the breakage of C1′-O4′ bond. While the newly formed ketone stabilizes very fast leading to a stable bond length of ∼1.28 Å, the C1′-O4′ distance diverges monotonically and eventually fluctuates between 2.0 and 2.75 Å. Figure 3A-III displays the behavior of the P-O5′ phosphate bond. It clearly demonstrates that an initial destabilization of this bond slowly recovers in time by dissipating energy away. The H4′ hydrogen abstraction alone does not lead to the breakage of the phosphate bond but weakens it. Figure 3A-III shows that the overall deformation of the nucleotide—it is critical to note the change in the angular correlation between the three sectors: the phosphate, the deoxyribose and the base. In the absence of any stacking of bases that is present in single- or double-stranded DNA, the base gets enough orientational freedom helping additional electron sharing between C1′ and N9 leading to a substantial change in angular correlation between these three sectors. In the following, we will see that the case is not similar for a single- or double-stranded DNA where there is stacking of bases. Electron density analysis provides further credence to the observed structural dynamics. Figure 4A-I and II exhibit that the electronic density distribution (i) increases along C4′ = O4′ bond revealing the ketone formation, (ii) ceases along C1′-O4′ bond revealing the breakage of this bond and (iii) increases along C1′-N9 bond revealing additional electron sharing.

### H4′ abstraction from single-stranded DNA

To describe the dynamics of H4′ hydrogen abstraction from a single-stranded DNA, we provide some representative structures from the reaction dynamics in Figure 2B. The dynamics of some key bond distances are shown in Figure 3B. We have observed that during the H4′ hydrogen abstraction phase, the P-O5′ bond is mostly affected (Figures 2B-II and 3B-III). As evident from Figure 3B-I, the hydrogen abstraction completes ∼70 fs. As C4′ loses H4′, it forms additional bonding interaction to satisfy the octet rule. From Figure 3B, we have observed that from 100 fs onwards, the C4′-O4′ bond distance starts decreasing and consequently, the C1′-O4′ bond distance starts increasing. This process ultimately leads to a consolidation toward C4′ = O4′ ketone formation and the weakening of the C1′-O4′ bond. From Figure 3B, we see that the C4′ = O4′ stabilizes to a bond length of ∼1.27 Å, while the C1′-O4′ bond distance fluctuates ∼1.7 Å. This is to note that as the C4′-O4′ bond strengthens toward forming a double bond, C1′-O4′ bond does not completely disrupt as we have seen in the case of the nucleotide. The absence of any orientational freedom of the guanine base restricts C1′ forming additional electron sharing with N9 of the imidazole ring of the guanine moiety as this involves π-electron delocalization that requires appropriate rotational symmetry. The C1′-N9 bond strengthens only partially; the bond distance reduces from 1.47 to 1.42 Å (Figure 2B-I and IV). Interestingly, in the backbone, we do not see any DNA cleavage that requires the breakage of the either P-O3′ or P-O5′ bonds or any of the O3′-C3′ or O5′-C5′ bonds. Immediately after the abstraction of H4′, both P-O3′ and P-O5′ bonds are found to be affected significantly and fluctuated strongly. However, as can be seen from Figure 3B-III, in the absence of any secondary reaction, these bonds stabilize themselves over time, intuitively by transferring the energy away over the backbone. The P-O5′ bond length stabilizes ∼1.66 Å, decreasing from a maximum of 1.76 Å while the P-O3′ bond length stabilizes to ∼1.7 Å from a maximum of 1.77 Å. Thus, out of these two, the P-O3′ bond is destabilized the most as is also found in the experiment (4,17). Our simulation data reveal that, for a H4′ hydrogen abstraction alone, it is unlikely to have any DNA cleavage through the breakage of any the P-O or C-O bonds at the phosphodiester linkages at P-O3′-C3′ or P-O5′-C5′. However, a closer look at the structural trajectory reveals that in the immediate aftermath of H4′ hydrogen abstraction, the C3′-O3′ and C5′-O5′ bonds also suffer destabilization as the released energy dissipates through the backbone. This is consistent with literature as it reveals that C4′ radical DNA may hydrolyze at C3′ or C5′ leading to the breakage of C3′-O3′ or C5′-O5′ and the release of DNA scission fragments with 3′- and 5′-phosphate termini, respectively (5,6). The structural trajectories generated for H4′ hydrogen abstracted C4′ radical DNA in solution (Figure 2B) will allow one to identify suitable conformation for QM/MM simulation of hydrolysis reactions at the phosphodiester bonds and will be of great interest. The feature that, without any secondary interaction, the DNA lesion partly recovers from the initial damage is in accordance with experimental observation (21) and validates the QM/MM simulation protocol employed to mimic DNA oxidation. An inadequate theoretical description of the QM system might have resulted in the dissociation of these bonds.

### H4′ abstraction from double-stranded DNA

The H4′ hydrogen abstraction trajectory from a double-stranded DNA is shown Figures 2C and 3C. The H4′ abstraction dynamics and subsequent changes in the double-stranded DNA is very much similar to those obtained in the case of a single-stranded DNA. The change in the C4′-O4′ bond distance from 1.41 Å (before hydrogen abstraction) to 1.27 Å (after hydrogen abstraction) along with weakening of C1′-O4′ bond (1.42–1.61 Å) signals the partial formation of a ketone (C4′ = O4′). The P-O3′ and P-O5′ bonds are strongly affected in the aftermath of the H4′ hydrogen abstraction but they partially recover in time. The main differences are coming from the base-pair hydrogen bonding that restricts the rotation of the base further. Consequently, we have observed that in the end, less damage occurs to the C1′-O4′ and the C1′-N9 bonds fully recovers the immediate damage with time compared to the case of the single-stranded DNA. Figures 2C-I–IV and 3C-III demonstrate that in the absence of any secondary reaction with water or molecular oxygen, as proposed in analyzing the experimental data (17), these 3′ and 5′ DNA backbone bonds could partially recover the initial damage by dissipating energy over the DNA backbone; P-O3′ sustains the maximum damage (Figure 3C-III) as noted in experiment (4,17). The structural trajectory reveals that H4′ hydrogen abstraction also destabilizes C3′-O3′ and C5′-O5′ bonds as the released energy dissipates through the backbone. Further reactions, like a hydrolysis of the radical C4′ DNA at C3′ or C5′, could lead to the breakage of C3′-O3′ or C5′-O5′ and the cleavage of the DNA with 3′- or 5′-phosphate termini as predicted in literature (4–6). In Figure 4B-I and II, we display the electron density distribution of the selected frames, which reveal the stabilization of C4′ radical with partial formation of a C4′ = O4′ ketone and reduction of electron density along the C1′-O4′ bond. But, we see very little change along C1′-N9 bond, revealing the absence of additional electron sharing in the presence of constraints for the rotational motion of the base in a double-stranded DNA. In the absence of any double strand break from a single hydrogen abstraction reaction at C4′, we then studied the consequences of targeted the oxidation of one of the H5′ hydrogen.

### H5′ abstraction from double-stranded DNA

In Figure 2D, we present selected frames from H5′ abstraction dynamics by OH radical. The key bond dynamics are shown in Figure 3D. As can be seen from these two figures, both the C5′-O5′ and P5′-O5′ bonds get strongly affected during the hydrogen abstraction process. Eventually, C5′ forms C5′ = O5′ ketone with the bond distance reducing from 1.41 Å (before hydrogen abstraction) to 1.26 Å (after hydrogen abstraction). From Figure 3D-III, we see that as C5′ = O5′ double bond stabilizes to 1.26 Å, the P-O5′ bond destabilizes further with the distance between the atoms increasing to ∼2.0 Å (Figure 2D-IV). Electron density calculation on the frame shown in Figure 2D-IV reveals that the P-O5′ bond is completely broken at this point leading to the scission of the DNA strand. This is to be noted that the structural constraints present in a double-stranded DNA has no effect in H5′ abstraction-induced DNA scission. On top, H5′ is one of the most exposed hydrogen of a single- or double-stranded DNA. However, in order to achieve cleavage of a double-stranded DNA, it requires a complementary scission of the other strand of the duplex that can be similarly caused by another OH radical-induced H5′ abstraction. This is fully consistent with experimental observation where radical OH-induced DNA cleavage is found to be dependent on the perceived concentration of radical OH (5).

## DISCUSSION

In summary, we show that by employing the hybrid QM/MM molecular dynamics simulation protocol to single- and double-stranded DNA together in explicit water, it is possible to study the molecular mechanism of DNA damage reaction pathways and generate structural trajectories from hydrogen abstraction by an OH radical. We exhibit that the QM/MM tool could provide the required insight into the molecular mechanism of DNA damage reaction pathways that is otherwise quite difficult to find experimentally. Our results show that the abstraction of H4′ from double-stranded DNA causes moderate disruption of the deoxyribose ring through partial formation of a ketone between C4′ and O4′ and permanent weakening of the C1′-O4′ bond. In the absence of any rotational degrees of freedom of the base with respect to the deoxyribose, the initial strong fluctuations on the deoxyribose ring have been found to recover with time as the energy dissipates through the backbone. During this latter process, both the P-O3′ and P-O5′ bonds experience strong perturbations with P-O3′ destabilized the most. However, as the energy dissipates over the DNA backbone, these two bonds fluctuate in the range of 1.65 to 1.7 Å, causing only a permanent weakening of these bonds. Similar fluctuations and weakening are also observed at the phosphodiester backbone bonds C3′-O3′ and C5′-O5′. These results confirm the existing experimental assertion that H4′ hydrogen abstraction alone is not expected to lead to direct DNA cleavage but make the radical DNA vulnerable to secondary reactions with molecular oxygen or water at the phosphodiester backbone bonds C3′-O3′ and C5′-O5′ and lead DNA scission with 3′ or 5′ phosphate termini. Structural trajectories generated here for the C4′ radical DNA in solution would allow us to study its secondary reactions with water and would be of major interest. However, hydrogen abstraction from C5′ can lead to the breakage of P-O5′ bond and permanent cleavage of the DNA backbone and strand scission at 5′ end. Thus, targeting H5′ abstraction could be a suitable strategy to design DNA cleaving molecules or anticancer agents from pure oxidative interactions.

## Supplementary Material

Supplementary Data
